# Tightrope Walking: Using Predictors of 25(OH)D Concentration Based on Multivariable Linear Regression to Infer Associations with Health Risks

**DOI:** 10.1371/journal.pone.0125551

**Published:** 2015-05-27

**Authors:** Ning Ding, Keith Dear, Shuyu Guo, Fan Xiang, Robyn Lucas

**Affiliations:** 1 Faculty of Health, University of Canberra, Canberra, ACT, 2601, Australia; 2 National Centre for Epidemiology and Population Health, Research School of Population Health, The Australian National University, Canberra, ACT, 2600, Australia; 3 Duke Global Health Institute, Duke Kunshan University, Kunshan, Jiangsu, 215316, China; 4 Telethon Kids Institute, University of Western Australia, Perth, WA, 6009, Australia; University of Leicester, UNITED KINGDOM

## Abstract

The debate on the causal association between vitamin D status, measured as serum concentration of 25-hydroxyvitamin D (25[OH]D), and various health outcomes warrants investigation in large-scale health surveys. Measuring the 25(OH)D concentration for each participant is not always feasible, because of the logistics of blood collection and the costs of vitamin D testing. To address this problem, past research has used predicted 25(OH)D concentration, based on multivariable linear regression, as a proxy for unmeasured vitamin D status. We restate this approach in a mathematical framework, to deduce its possible pitfalls. Monte Carlo simulation and real data from the National Health and Nutrition Examination Survey 2005–06 are used to confirm the deductions. The results indicate that variables that are used in the prediction model (for 25[OH]D concentration) but not in the model for the health outcome (called instrumental variables), play an essential role in the identification of an effect. Such variables should be unrelated to the health outcome other than through vitamin D; otherwise the estimate of interest will be biased. The approach of predicted 25(OH)D concentration derived from multivariable linear regression may be valid. However, careful verification that the instrumental variables are unrelated to the health outcome is required.

## Introduction

Evidence suggests that higher vitamin D status is associated with a decreased risk of various cancers and chronic diseases, beyond its essential role in bone health [1]. In epidemiologic studies examining vitamin D deficiency as a risk factor for disease, vitamin D status is measured as the serum concentration of 25-hydroxyvitamin D (25[OH]D) [2]. However, for large-scale health surveys, measuring 25(OH)D concentration for each participant is not always feasible because of the logistics of blood collection and the costs of vitamin D testing.

To overcome this problem, it has been common to use an indicator of vitamin D status, such as latitude or level of solar ultraviolet radiation, as a proxy for 25(OH)D [3–5]. Recent studies have however shifted toward using multivariable linear regression models to predict 25(OH)D concentration [6]. Briefly, the relationship between measured 25(OH)D concentration and determinants is identified by multivariable linear regression within a subset of participants. Based on the estimates derived, the unobserved 25(OH)D concentration is predicted and then the predicted value is used to analyze the association with the health outcome of interest. This methodology has been used to demonstrate a protective association between vitamin D sufficiency and risk of various cancers [6–9].

Here we firstly restate this approach in a simple but general mathematical framework. We deduce that the variables, called instrumental variables, which appear only in the multivariable linear regression for the prediction, but not in the health outcome equation, are vital for the correct identification of the association between 25(OH)D concentration and the health outcome. If the instrumental variables are in fact associated with the health outcome, and therefore are invalid as instruments, the estimated effect may be significantly biased. We then use Monte Carlo simulation and real data from the National Health and Nutrition Examination Survey (NHANES) to demonstrate this potential bias. Overall, we highlight problems that may occur when using this methodology to gain a better understanding of the potential for misleading results due to the use of invalid instrumental variables.

### Predictors of 25(OH)D Concentration Based on Linear Regression

Recent studies using predicted 25(OH)D concentrations based on multivariable linear regression to investigate associations between vitamin D status and health outcomes, such as incidence of cancers, diabetes, or Crohn’s disease are summarized in Table 1.

**Table 1:** Summary of papers employing predicted 25(OH)D score to examine associations between vitamin D status and health outcomes.

Reference	Health outcome	Statistical model in *Stage II*	Instrumental variables(s)
Giovannucci et al, 2006 [6]	Cancer incidence and mortality	Cox	Geographical residence, Dietary vitamin D intake, vitamin D supplements, Race
Ng et al, 2009 [9]	Colorectal cancer	Cox	Geographical region
Liu et al, 2010 [15]	Type 2 diabetes	Cox	Month of blood sampling, total vitamin D intake, physical activity score, smoking status, total energy intake, BMI^a^
Jimenez et al, 2012 [19]	Tooth loss and periodontitis	Cox	UVB radiation flux at residence, dietary and supplemental intake of vitamin D
Gilbert et al, 2012 [29]	Risk factors for prostate cancer (PSA level, BMI, Family history of prostate cancer)	Linear and Logistic	Sun exposure, dietary intake, Anthropometric, clinical and demographic factors^b^
Liu et al, 2013[16]	Endometrial cancer	Cox	Vitamin D intake from food, vitamin D intake from supplements, UVB flux based on state of residence, physical activity, alcohol intake
Ananthakrishnan et al, 2012 [18]	Crohn's disease	Cox	Dietary and supplemental vitamin D intake, exposure to sunlight, race, regional ultraviolet-B radiation intensity
Harris et al, 2013 [17]	Endometriosis	Cox	Race, geographical region, season of blood draw, dietary vitamin D intake
Joh et al, 2013 [8]	Renal cancer	Cox	UVB radiation flux at residence, dietary and supplement intake of vitamin D, postmenopausal hormone use

^a^ Adjusted for waist circumference in *Stage II*;

^b^ Since backwards stepwise regression was employed, the instrumental variables used varied across regressions.

This is a two-stage, two-dataset method.

#### Stage I (using a subset of the main dataset)

The determinants of 25(OH)D concentration are identified based on the analysis of a subset of the full dataset which is assumed to be representative of the whole sample. Measured 25(OH)D concentration is available in this subset. The following model is estimated:

D=ax+dz+e, (1)

where *x* and *z* are the possible determinants of 25(OH)D concentration, *D*, and *e* is the error term. For the sake of simplicity, *x* and *z* are assumed to be single variables, however, the derivation can be generalized to the case of multiple regression by allowing *x* and *z* to be vectors. Furthermore, either *x* or *z* is assumed to be uncorrelated with *e*, and so the parameter estimates, $\hat{a}$ and $\hat{d}$ are unbiased.

#### Stage II (Using the Main Dataset)

In the Full Dataset, the Missing 25(OH)D Concentration (“Score”) Is Predicted as

$$\hat{D}=\hat{a}x+\hat{d}z.$$ (2)

The predicted 25(OH)D score, $\hat{D}$, is used as a proxy for the real 25(OH)D concentration, even for those with measured values.

Here we use the Cox proportional hazard model in *Stage II* as an example, as it has been widely used in the research-to-date that has employed this approach. The form of the model is:

$$H\left(t\right)=h_{0}\left(t\right)\;\text{exp}\left(\alpha x+\beta y+\theta \hat{D}\right),$$ (3)

where *H*(*t*) and *h*_0_(*t*) are the hazard rate and the baseline hazard respectively.

In this example, *x* is a common covariate in *Stage I* and *II* and in practice includes, for example, age and sex; *y* appears only in *Stage II* and is a factor associated with the health outcome but not with 25(OH)D concentration, for example, vegetable and fruit intake and family history of the disease. The variable *z* is excluded from *Stage II*. The exclusion of *z* (hereafter, referred to as an “instrumental variable”) from Eq (3) is necessary to avoid a problem with multicollinearity that arises because $\hat{D}$ is a linear combination of *x* and *z* (using the predictive model derived in *Stage 1*) [10]. If *x*, *z* and $\hat{D}$ are all introduced, most computer software packages will drop one of them and the estimation will be equivalent to that of Eq (3). Among papers utilizing this approach, variables such as geographic residence, vitamin D intake, race, alcohol intake and variants of genes, are used as instrumental variables Table 1.

There are two important requirements of a valid instrumental variable. First, it must be significantly associated with the 25(OH)D concentration, *D*, conditioning on *x*; and, second it should not be a risk factor for the disease of interest except through its effect on *D*. If the first requirement is not satisfied, the estimated standard error of the association between the 25(OH)D score and the health outcome may be very large and/or the effect estimate will be inconsistent. However, this problem is easily detected and avoided in practice, since whether *z* is statistically significant in *Stage I* can be easily checked. We will not discuss this problem further.

We examine the effect of violation of the second requirement, that an instrumental variable should not be a risk factor for the disease except through its effect on *D*. This cannot be detected statistically and may result in bias in the estimate of interest.

Consider theoretically,

$$H\left(t\right)=h_{0}\left(t\right)\;\text{exp}\left(\alpha x+\beta y+\delta z+\theta \hat{D}\right);$$ (4)

**Fig 1:**
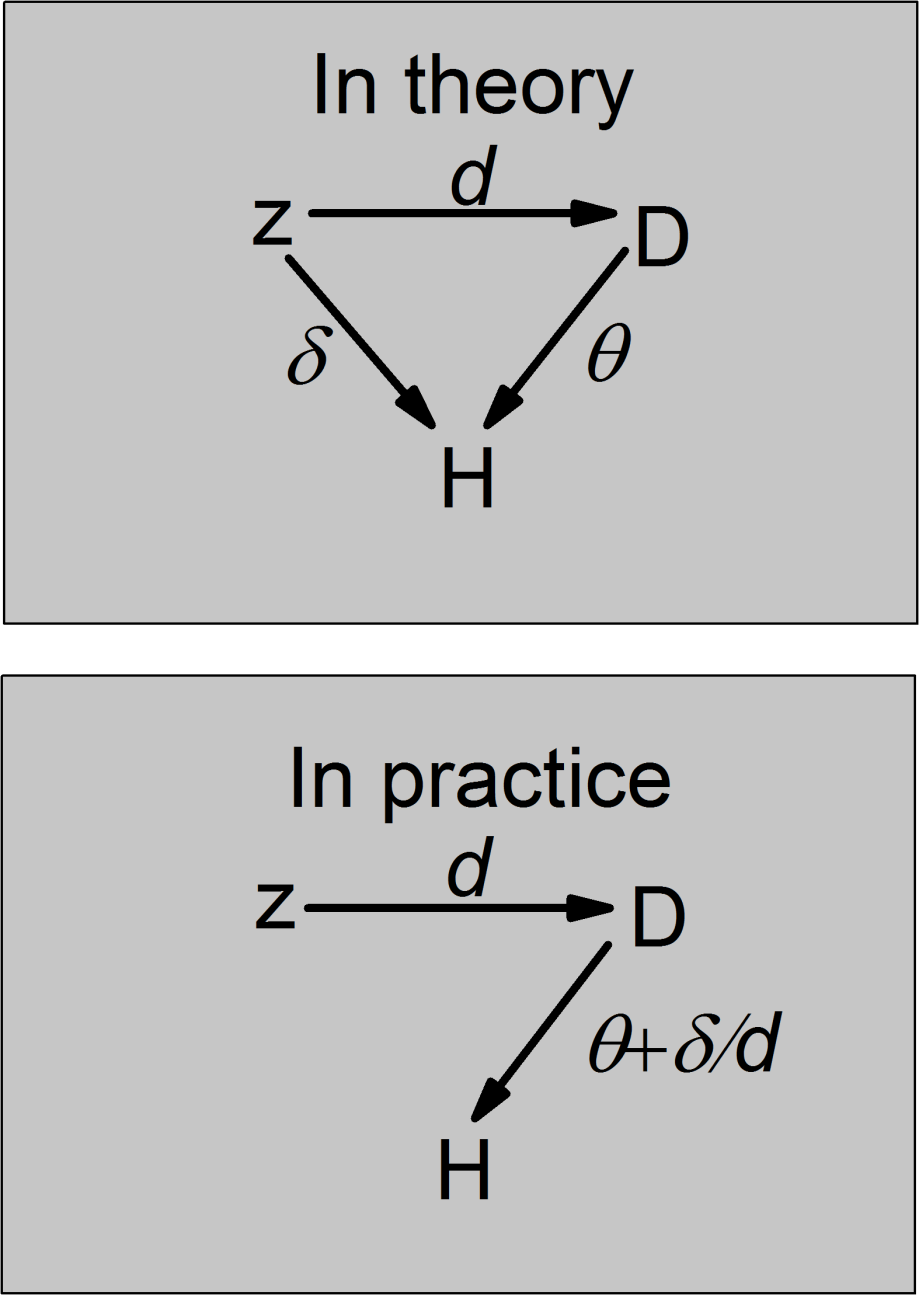
The effect of omitting *z* from the health outcome equation. In theory, the total effect of *z* on health outcome, H, is *δ* + *θd*. In practice, using *z* as an instrumental variable causes bias in the estimated effect of the 25(OH)D score, *D*, on the health outcome, because the direct effect of *z* on the health outcome is incorrectly captured as being mediated by *D*.

Here, *z* is employed as an instrumental variable although it is not valid, and Eq (3) is estimated. As demonstrated in S1 Appendix, the bias in the estimated effect of 25(OH)D concentration on the health outcome is $\delta /\hat{d}$ where $\hat{d}$ is the estimate of the association between measured 25(OH)D concentration and *z*. The intuitive explanation is illustrated in Fig 1. That is, the bias occurs because the direct association between *z* and the health outcome, *H*(*t*), forms part of the association between 25(OH)D concentration, *D*, and the health outcome, *H*(*t*). Thus, on the one hand, the bias is positively associated with *δ* (i.e. the larger the effect estimate of the association between *z* and the health outcome, the greater is the contribution of that association to the association of *z* with 25(OH)D, *D*). On the other hand, the stronger the association between *z* and *D*, the greater is the ‘proportion’ of the association between *z* and the health outcome that is working via the 25(OH)D concentration, and the smaller the ‘proportion’ that is the direct association between *z* and the health outcome. As a result, the bias is inversely associated with *d*.

These deductions can be generalized to generalized linear models (GLMs), such as linear, logistic, or Poisson regression [11,12]. In these cases, $\alpha x+\beta y+\theta \hat{D}$ is the linear predictor in the GLM framework.

In order to assess the empirical importance of correctly choosing the instrumental variables, we implemented Monte Carlo simulations to generate a series of virtual datasets as well as examining real data from NHANES 2005–2006 to examine the association between 25(OH)D concentration and systolic blood pressure.

## Methods and Materials

### Monte Carlo Simulation

#### Data Generating

The Monte Carlo design assumes the 25(OH)D concentration is generated according to the following linear equation

$$D=a_{0}+a_{1}x+d_{1}z_{1}+d_{2}z_{2}+e.$$ (5)

We set *a*_0_ = 0.1, *a*_1_ = 0.4, *d*_1_ = 0.2 and *d*_2_ = 0.3. The covariates, *x*, *z*_1_ and *z*_2_ are all drawn independently from the standard normal distribution, and the error term *e* from a uniform distribution between -0.5 and 0.5.

The hazard of the health outcome is defined as a function of *x*, y, *z*_1_ and *D*.

$$H\left(t\right)=h_{0}\left(t\right)\;\text{exp}\left(\alpha_{1}x+\beta y+\delta_{1}z_{1}+\theta D\right),$$ (6)

We set *α*_1_ = 0.1, *β* = 0.3, *δ*_1_ = 0.4, *θ* = 0.5 and *h*_0_(*t*) = 1. The covariate *y* was drawn from the standard normal distribution independently.

The method for the generation of the survival time for the proportional hazard models is introduced in S2 Appendix. Furthermore, in order to show how the bias changes with either of *d*_1_ or *δ*_1_, *d*_1_ was set to change from -1 to 1 with a step size of 0.1 with *δ*_1_ fixed at 0.4; and *δ*_1_ was set to change from -1 to 1 with a step size of 0.1 with *d*_1_ fixed at 0.2. For each level of *d*_1_ and *δ*_1_ 1,000 datasets with 5,000 observations were generated. Note, *z*_2_ is always a valid instrumental variable; however only when *δ*_1_ = 0, is *z*_1_ valid because only in this case is *z*_1_ not an explanatory factor for the health outcome.

#### Estimating

When estimating, three specifications were employed. In Specification I, only ***z***_**1**_ is used as an instrumental variable. In other words, ***z***_**1**_ is included in the equation to predict 25(OH)D score, but not in the equation testing the association with the health outcome. In Specification II, both **z**_**1**_ and **z**_**2**_ are used as instrumental variables, and in Specification III, only **z**_**2**_ is used as an instrumental variable. Hence, only Specification III is correct and the estimates arising should be unbiased.

The means of the estimated association with the health outcome over the 1,000 datasets for each level of *d*_1_ and *δ*_1_ were compared with each actual value.

### Source of Real Data

The methodology for NHANES 2005–06 is well-described elsewhere [13]. Briefly, the survey is designed to assess the health and nutritional status of adults and children in the United States. NHANES 2005–06 was the seventh NHANES and included more than 10,000 participants from 30 sites across the United States. The data from this survey are used here as 25(OH)D concentration is available on a large sample size.

We chose to examine the association between 25(OH)D concentration and systolic blood pressure (as the health outcome of interest) as an example. It is unclear that there is any causal relationship between 25(OH)D level and blood pressure. Nevertheless the association is used here to demonstrate the potential bias caused by the use of an invalid instrumental variable. Exclusion of data from participants with missing values for 25(OH)D concentration or with fewer than three readings of systolic blood pressure and children (aged<18 years), resulted in a final sample of 4,002 participants. The missing data for 25(OH)D concentration and systolic blood pressure were missing at random [14]; specifically, missingness was associated with gender and overweight or obesity status

### Statistical Methods

The dependent variable, systolic blood pressure, is continuous; thus ordinary least squares regression (OLS) is used for *Stage II*. This also provides an opportunity to test the generalizability of our theoretical analysis.

First, we estimated the association between 25(OH)D concentration and systolic blood pressure. The results were used as the benchmark against which to check whether there was bias caused by use of invalid instrumental variables. Covariates included age, sex (reference category = ‘Female’), and overweight or obesity status (reference category = ‘BMI≤25’).

Next, we developed a predictive model for 25(OH)D concentration using a multivariable OLS linear regression model (*Stage I*). The determinants of 25(OH)D concentration included sex, and overweight or obesity.

In *Stage II*, we used the same dataset but set all of the values of 25(OH)D concentration to be missing, and replaced these with a predicted 25(OH)D score derived from *Stage I*. We used OLS regression again, using sex and the predicted 25(OH)D score to assess the association between 25(OH)D score and systolic blood pressure (*Stage II*). Thus, the instrumental variable of interest was overweight or obesity status (included in *Stage I*, but not *Stage II*). There are many potential explanatory variables in both stages; however, use of only overweight/obesity is sufficient to illustrate the outcome of using an invalid instrumental variable.

All analyses were performed using Stata 11.

## Results

### Monte Carlo Simulation

The effect estimates for the different variables, based on virtual samples for all three specifications, are in Table 2. The results confirmed that valid instrumental variables are essential for the estimates to be unbiased. If the instrumental variable is invalid, the bias in the estimated effect of 25(OH)D on the health outcome (*θ*) can be large, even if all of the coefficients in these models are reasonably small. For example, when *d*_1_ and *δ*_1_ were 0.2 and 0.4 respectively, the bias was 2 (*δ*_1_/*d*_1_ = 0.4/0.2), with the estimated coefficient nearly five-fold higher than the “real” (pre-set) value of *θ* (see Specification I Table 2). For Specification II (using *z*_1_ and *z*_2_ as instrumental variables), the bias in the estimate of the association between 25(OH)D and the health outcome, (*θ*) remained significant (p<0.001) but was smaller. Only when the instrumental variable is valid are the estimates statistically identical to the pre-set values (Specification III).

**Table 2:** *Stage-II* estimates of the coefficient for each of the variables (x, y, D) included in the Cox proportional hazard models for different specifications, Monte Carlo simulations, sample size 5000, *d*_1_ = 0.2 and *δ*_1_ = 0.4.

Variable	Pre-set value	Estimated value	95% CI	*P*-value
Specification I^a^				
*x*	0.10	-0.68	(-0.74, -0.61)	<0.001
*y*	0.30	0.29	(0.26, 0.32)	<0.001
*D*	0.50	2.42	(2.27, 2.57)	<0.001
Specification II^b^				
*X*	0.10	-0.13	(-0.18, -0.09)	<0.001
*y*	0.30	0.27	(0.25, 0.30)	<0.001
*D*	0.50	1.02	(0.93, 1.10)	<0.001
Specification III^c^				
*x*	0.10	0.10	(0.05, 0.15)	0.003
*y*	0.30	0.30	(0.27, 0.32)	<0.001
*z*_1_	0.40	0.39	(0.36, 0.43)	<0.001
*D*	0.50	0.49	(0.40, 0.59)	<0.001

^a^ *z*_1_ is the only instrumental variable, but it is invalid;

^b^ *z*_1_ and *z*_2_ are the invalid and valid instrumental variables respectively;

^c^ *z*_2_ is the only instrumental variable, and it is valid. Specification III is correct.

**Fig 2:**
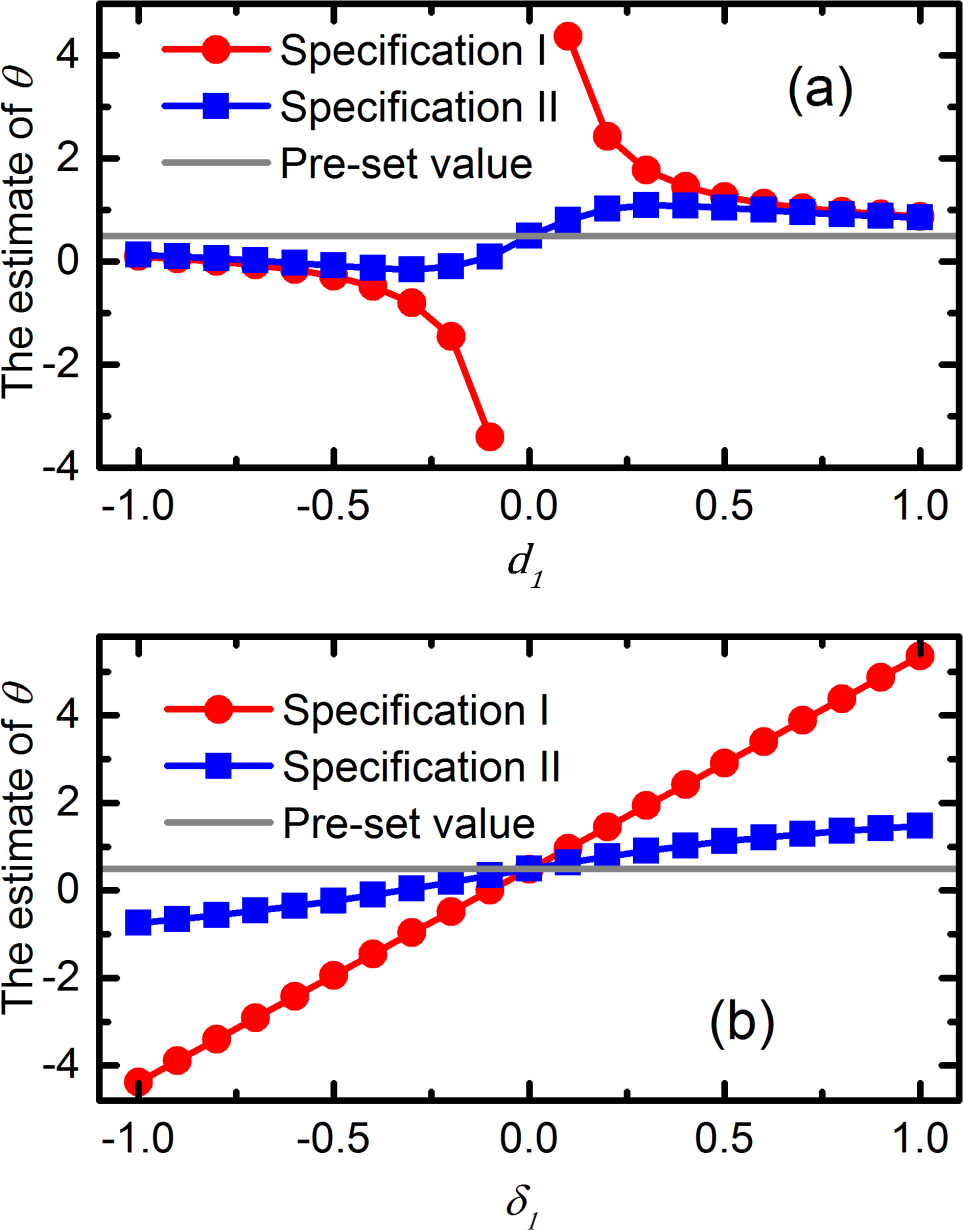
Estimates of the association between the 25(OH)D concentration and the health outcome (*ϴ*) according to the change in (a) *d*_1_, with *δ*_1_ = 0.4 and (b) *δ*_1_, with *d*_1_ = 0.2; sample size 5000, Specification I and II.

Fig 2(A) shows that the bias in the estimate of *θ* is inversely correlated with the magnitude of the association between *z*_1_ and 25(OH)D concentration. The results, when *d*_1_ is very close to 0, are not shown because the bias is theoretically infinite. The bias caused by the invalid instrumental variable decreases with increasing *δ*_1_. When *δ*_1_ is zero, *z* is a valid instrumental variable and the estimate is unbiased. Further, the comparison between Specification I and II suggests that, although the introduction of a valid instrumental variable improves the performance of the model, bias is still present.

Fig 2(B) shows that the estimate of *θ* increases almost linearly with the association between *z* and the health outcome. Again, bias is absent only if *δ*_1_ is equal to zero. A valid instrumental variable is helpful to identify the presence of an association between the 25(OH)D score and the health outcome, but the effect estimate is biased.

### Real Data

Table 3 presents summary data on the 4,002 adults in NHANES 2005–06 who had complete information on blood pressure and 25(OH)D concentration. Severe vitamin D deficiency was more common in participants who were overweight or obese compared to those of normal weight (p<0.001). Mean systolic blood pressure increased across categories of lower vitamin D status (p<0.001).

**Table 3:** Summary of characteristics according to vitamin D status (based on measured serum 25(OH)D concentration) among 4,002 adults of the National Health and Nutrition Examination Survey (2005–06), American adults who had three readings of systolic blood pressure.

	Severe deficiency<10 ng/mL	Mild deficiency10~20 ng/mL	Adequacy> = 20 ng/mL	*P-*value
Overall n(%)	365 (9.1)	1427 (35.6)	2210 (55.3)	
Gender^a^				0.14
Male, n (%)	141(7.3)	711(36.6)	1089(56.1)	
Female, n(%)	224(10.9)	716(34.7)	1121(54.4)	
Age (years), mean(SD)^b^	41.3 (18.3)	44.1 (19.0)	45.4 (18.8)	<0.001
Overweight or obesity status^a^				<0.001
BMI>25, n (%)	277(10.2)	1050(38.6)	1390(51.2)	
BMI≤25, n(%)	88(6.9)	377(29.3)	820(63.8)	
Systolic blood pressure (mmHg) mean(SD)^b^	124.1 (19.5)	123.4 (18.0)	120.7 (17.3)	<0.001

^a^ P values were derived from Kolmogorov-Smirnov tests;

^b^ P values for trend (two-sided) were derived from trend tests.

The results in the first column of Table 4 show that the measured 25(OH)D concentration was inversely associated with systolic blood pressure after controlling for age, sex, and overweight or obesity status (β = -0.16 (95% CI: -0.21, -0.11; p<0.001)). In addition, systolic blood pressure was positively and significantly associated with overweight status: when BMI was greater than 25, the systolic blood pressure was higher by 3.13 (95% CI: 2.08, 4.18; p<0.001) mm Hg.

**Table 4:** Multivariable association between measured 25(OH)D concentration and systolic blood pressure; determinants of 25(OH)D concentration (*Stage I*); and multivariable association between predicted 25(OH)D score and systolic blood pressure (*Stage II*) among 4,002 adults of the National Health and Nutrition Examination Survey (2005–06), US, OLS.

Variable	Systolic blood pressure			*Stage I*: measured 25(OH)D concentration			*Stage II*: Systolic blood pressure		
	Coefficient	95% CI	*P*-value	Coefficient	95% CI	*P*-value	Coefficient	95% CI	*P*-value
Measured 25(OH)D concentration	-0.16	(-0.21, -0.11)	<0.001						
Predicted 25(OH)D score							-1.15	(-1.48, -0.82)	<0.001
Age	0.42	(0.40, 0.45)	<0.001	0.02	(0.01, 0.04)	<0.001	0.45	(0.42, 0.47)	<0.001
Male (vs. female)	3.71	(2.74, 4.67)	<0.001	-0.28	(-0.86, 0.30)	0.35	3.43	(2.45, 4.41)	<0.001
Overweight or obesity (BMI>25 vs≤25)	3.13	(2.08, 4.18)	<0.001	-3.16	(-3.79, -2.53)	<0.001			
Constant	102.63	(100.80, 104.45)	<0.001	22.89	(21.05, 23.74)	<0.001	130.19	(124.55, 132.45)	<0.001
Adjusted R^2^	0.24			0.02			0.24		

The results of the OLS linear regression in *Stage I* based on the measured 25(OH)D concentration, shown in Column (2) of Table 4, suggested that overweight or obesity status was a significant predictor of 25(OH)D concentration. Compared to a BMI of ≤25, a BMI of greater than 25 was associated with a 3ng/ml lower measured 25(OH)D concentration (β = -3.16; 95% CI: -3.79, -2.53 ng/mL; p<0.001). The *Stage II* estimate for the association between the predicted 25(OH)D score and systolic blood pressure, where overweight or obesity status was employed as the instrumental variable, was β = -1.15 (95% CI: -1.48, -0.82; p<0.001) (Column (3) of Table 4). This is significantly different from the estimate based on the measured 25(OH)D concentration (Column (1) of Table 4) (p<0.001).

In addition, because the coefficient of the association between overweight or obesity status and systolic blood pressure was 3.13, and that between overweight or obesity status and 25(OH)D concentration was -3.16 (See Column (1) and (2) of Table 4 respectively), the bias is theoretically -0.99 (= 3.13/-3.16) when overweight or obesity status is used as the instrumental variable. Thus, the estimated effect of the predicted 25(OH)D score on systolic blood pressure is β = -1.15 (= [-0.16] +[-0.99]), that is, the sum of the estimated effect of the measured 25(OH)D concentration in Column (1) of Table 4 and the bias. This is statistically identical with the estimate in column (3) of Table 4, -1.15 (95% CI: -1.48, -0.82).

## Discussion

The results indicate that the prediction of 25(OH)D concentration based on multivariable linear regression may be correct, but care needs to be taken when applying this methodology. Even if only one of the instrumental variables used is invalid, the estimates of the association between 25(OH)D concentration and the health outcome will be unreliable. It should be noted that the second requirement of a valid instrument variable, that it should not be a risk factor for the disease, cannot be test mathematically or statistically and can only be judged according to biological findings from past research. Thus, the reasons for the choice of instrumental variables should be discussed, and the lack of correlation with the health outcome confirmed. Previous studies using this methodology have not provided an adequate consideration of the potential biases that could occur. For example, several papers used variables such as physical activity, BMI, smoking status, alcohol intake and race as instrumental variables, despite substantial evidence these factors are strongly associated with many diseases, including the outcomes of interest [6,15–18]. Vitamin D intake has also been used as an instrumental variable [8,19], but may also be associated with disease risk as a marker of a healthier lifestyle and thus lower disease risk [20].

In some studies, stratification by a potential confounder, or meta-analysis of findings have been used to indicate a greater likelihood of a “real” finding. However, a stratified analysis cannot demonstrate that the results are “correct” or robust. For example, where BMI is used in the predictive model for 25(OH)D score, then the effect estimate of 25(OH)D score on the health outcome, e.g. digestive cancer, may be compared across strata of BMI. Higher BMI is a known risk factor for digestive cancer and is therefore an invalid instrumental variable. In this case, if the effect estimates from the two strata are the same or similar, then the conclusion may be that the association between 25(OH)D score and the health outcome is the same for both strata, or, alternatively, that the bias caused by the invalid instrumental variable plus the real association is the same for both strata. But it is not possible to distinguish between these two possible conclusions. Similarly, meta-analysis does not help although it is useful to estimate the summary effects over a number of previous studies particularly when the sample size in any single study is insufficient. If all of the individual studies use invalid instrumental variables, all of the effect estimates are biased, and the weighted average of these biased estimations will be similarly biased.

Most recently, variants of genes that affect 25(OH)D synthesis or substrate availability (e.g. *CYP2R1*, *GC* and *DHCR7*) have been used as instrumental variables either individually or through creation of a genetic score that acts as a proxy for long-term 25(OH)D levels [21]. This method does not predict 25(OH)D levels per se, but may be more disease-relevant than a single 25(OH)D measurement for which intraclass correlation coefficients range from 0.42–0.72 between 2 direct measures taken 2–14 years apart [6,22–24]. The substrate from which vitamin D is synthesised is 7-dehydrocholesterol (7-DHC) located in epidermal cells of the skin. The *DHCR7* gene encodes the enzyme 7-DHC reductase and both 7-DHC and 7-DHC reductase are part of the cholesterol biosynthesis pathway. Using a genetic synthesis score, a recent meta-analysis showed a modest association between higher genetically instrumented 25(OH)D concentration and lower systolic blood pressure A valid instrument has an effect on the outcome only through the factor that it is a proxy for, in this case 25(OH)D concentration. In the recent study, the synthesis score was highly correlated with measured 25(OH)D concentration, but also had an overall association with higher serum total cholesterol (p = 0.04), suggesting a possible separate pathway of effect of this genetic score on higher systolic blood pressure. Thus genetic 25(OH)D scores should also be used as instrumental variables with caution, given the pleiotropic effects of some vitamin D pathway genes, e.g. *GC* and its association with lipid metabolism, inflammation and metabolic feedback loops.

In practice, it is common to generate a dichotomous variable based on the predicted score to categorize participants as suffering from vitamin D deficiency or not, and this further complicates the situation. In this situation, the bias caused by use of an invalid instrumental variable will be further distorted by the distribution of the predicted 25(OH)D score. The direction of the bias cannot be determined theoretically.

The method discussed here is similar to the method of Two-Stage Least Squares (2SLS) which is widely used to estimate causal relationships in economics [25,26]. Differences between the two methods include that 25(OH)D concentration is available in the main data for Stage 1, but not Stage II, while 2SLS usually uses the same dataset in both stages. The 2SLS method aims to solve the bias caused by omitted confounders; an instrumental variable can be used only if it: 1) has a strong association with the variable (exposure) of interest; and 2) is not an independent risk factor for the outcome. These two criteria also apply for the methodology using a predicted 25(OH)D score.

Although applying the predicted 25(OH)D score method to identify the association between 25(OH)D concentration and health outcomes is not straightforward, there are clinical applications for predicted data. Recently there have been large increases in vitamin D testing in several countries due to concern about possible widespread vitamin D deficiency and purported links to a wide range of health risks [27], with considerable costs to healthcare systems [28]. One solution to reduce unnecessary tests is to predict those who are at high risk of vitamin D deficiency using available data, and test only these people. However, when predicted levels are used in large-scale epidemiological studies seeking to clarify links between vitamin D status and disease risks, there is considerable risk of bias in the estimates of effect arising from incorrect specification of an instrumental variable. This must be fully considered and discussed in studies using this methodology.

## Supporting Information

S1 Appendix The mathematical framework for a predictive model of 25(OH)D concentration, based on multivariable linear regression. (DOCX)

S2 Appendix Generating virtual survival times. (DOCX)
