# Supplementary material for: Tightrope Walking: Using Predictors of 25(OH)D Concentration Based on Multivariable Linear Regression to Infer Associations with Health Risks
Source: PLoS One. 2015 May 27;10(5):e0125551. doi: 10.1371/journal.pone.0125551 (PMC4445919; doi:10.1371/journal.pone.0125551)
Supplement: S1 Appendix — (DOCX) [file pone.0125551.s001.docx]

## S1 Appendix

**The mathematical framework for a predictive model of 25(OH)D concentration, based on multivariable linear regression**

The 25(OH)D concentration is a linear function of variables $x$, $z$ and the error term as follows

| $D=ax+dz+e,,$ | (A.1) |
| --- | --- |

Theoretically, the relationship between 25(OH)D concentration (D) and the health outcome is assumed to be:

| $H\left( t \right)=H_{0}\left( t \right)\exp\left( \Gamma\right)=H_{0}\left( t \right)\exp\left( \alpha x+\beta y+\delta z+\theta D \right),$ | (A.2) |
| --- | --- |

where $y$ is the determinant of health outcome but not of the 25(OH)D concentration. Substitute A.1 into A.2, we have.

| $H\left( t \right)=H_{0}\left( t \right)\exp[(\alpha+a\theta)x+\beta y+(\delta+\theta d)z+\theta e].$ | (A.3) |
| --- | --- |

In practice, the 25(OH)D concentration is predicted based on the regression (A.1) and let us assume the estimates are all unbiased and consistent. $\hat{D}=ax+dz$ (*Stage I*). However, in *Stage II*, the health outcome equation is mis-specified as

| $H\left( t \right)=H_{0}\left( t \right)\exp\left( \Gamma\right)=H_{0}\left( t \right)\exp\left( \alpha x+\beta y+\theta\hat{D} \right),$ | (A.4) |
| --- | --- |

Thus,

| $H\left( t \right)=H_{0}\left( t \right)\exp[(\hat{\alpha}+a\hat{\theta})x+\beta y+\hat{\theta}dz].$ | (A.5) |
| --- | --- |

Comparing the coefficients before $z$ in equation (A.3) and (A.5), we have

| $\delta+\theta d=\hat{\theta}d.$ | (A.1) |
| --- | --- |

Thus, $\hat{\theta}=\theta+\delta/d$ and the bias in the estimate of interest is $\delta/d$.

Further, comparison of the coefficients before $x$ in equation (A.3) and (A.5), shows

| $\alpha+a\theta=\hat{\alpha}+a\hat{\theta}.$ | (A.2) |
| --- | --- |

Then, $\hat{\alpha}=\alpha+a\left( \theta-\hat{\theta} \right)=\alpha-a\delta/d$ and the bias is $-a\delta/d$. Thus, invalid instrumental variables bias the estimates of the covariates which appear in both stages too.
