# Supplementary material for: Tightrope Walking: Using Predictors of 25(OH)D Concentration Based on Multivariable Linear Regression to Infer Associations with Health Risks
Source: PLoS One. 2015 May 27;10(5):e0125551. doi: 10.1371/journal.pone.0125551 (PMC4445919; doi:10.1371/journal.pone.0125551)
Supplement: S2 Appendix — (DOCX) [file pone.0125551.s002.docx]

## S2 Appendix

**Generating virtual survival times**

Since all of the variables are assumed to be time-invariant, the survival function of the proportional hazards model is given by

| $S\left( t \vert X,Y,D \right)=exp(-H_{0}(t)exp(\Gamma))$ | (B.1) |
| --- | --- |

where $H_{0}\left( t \right)=\int_{0}^{t} h_{0}\left( u \right)du$ is the cumulative baseline hazard function. Thus, the survival time, denoted by $T$, is in fact a random variable with distribution function $S$. So, $U=S(T)$ follows a uniform distribution on the interval from 0 to 1. Then

| $U=\exp\left( -H_{0}\left( T \right)\exp\left( \Gamma\right) \right).$ | (B.2) |
| --- | --- |

If the baseline hazard $h_{0}\left( t \right)>0$ for all $t$, then $H_{0}$ is invertible and the survival time $T$ of the proportional hazard model can be expressed as:

| $T=H_{0}^{-1}(-ln(U)\times exp(-\Gamma)).$ | (B.3) |
| --- | --- |

With the help of equation (B.3), the random variable of survival time can be generated.

If the baseline hazard rate is a constant, $h_{0}\left( t \right)=\lambda$ (the exponential model), then $H_{0}\left( t \right)=\int_{0}^{t} \lambda du=\lambda t.$ So, $H_{0}^{-1}\left( t \right)=\lambda^{-1}t.$ Thus, $T=-\lambda^{-1}ln(U)\times exp(-\Gamma)$.
